# A Cause of Bilateral Chylothorax: A Case of Mesothelioma without Pleural Involvement during Initial Diagnosis

**DOI:** 10.1155/2015/962504

**Published:** 2015-09-16

**Authors:** Ercan Kurtipek, Meryem İlkay Eren Karanis, Nuri Düzgün, Hıdır Esme, Mustafa Çaycı

**Affiliations:** ^1^Department of Chest Diseases, Konya Training and Research Hospital, 42090 Konya, Turkey; ^2^Department of Pathology, Konya Training and Research Hospital, 42090 Konya, Turkey; ^3^Department of Thoracic Surgery, Konya Training and Research Hospital, 42090 Konya, Turkey; ^4^Department of Nuclear Medicine, Konya Training and Research Hospital, 42090 Konya, Turkey

## Abstract

Chylothorax is characterized by fluid accumulation in the pleural cavity containing chylomicrons due to disruption of lymphatic drainage in the thoracic ductus and development of chylothorax. A 60-year-old male patient presented to our clinic with shortness of breath and displayed bilateral pleural effusion and diffuse mediastinal lymph nodes in his computed chest tomography images. There were no thickening and nodular formation on the pleural surfaces. PET-CT showed no pathological FDG uptake. Thoracentesis showed a chylous effusion. Drainage reduced during monitoring could not be stopped; therefore, surgical intervention was considered. The patient underwent right thoracotomy. There were no pathological findings in the parietal and visceral pleura during the surgery. Initially lymphoma was considered. Perioperative samples were collected from the mediastinal lymph node. The pathology analysis reported metastasis of malignant mesothelioma. Evaluation of a repeated chest computed tomography showed nodular formations on the pleural surfaces. Mediastinal lymph nodes compressed the ductus thoracicus, resulting with chylothorax. The present case, with malignant mesothelioma, bilateral chylothorax, and mediastinal lymph node without any pleural involvement during initial diagnosis, is rare and will hence contribute to the literature.

## 1. Introduction

Formation of chylous in the pleural space due to a damage or blockage of the thoracic duct is called chylothorax. It can result from tumors, lymphatic involvement, direct invasion, or tumor embolus, leading to spontaneous chylothorax. Lymphoma is the most common cause of nontraumatic chylothorax [1]. The majority of them develop secondary to obstruction of the lymphatic pathways from mediastinal lymphomas. Other malignancies with mediastinal involvement and infectious diseases may be associated with chylothorax. Granulomatous diseases are also associated with chylothorax [2]. Malignant pleural mesothelioma (MPM) is a locally aggressive tumor with a very poor prognosis, where exposure to asbestos is the major etiology. Rare causes of MPM include radiotherapy, tuberculosis, and chronic empyema. Common findings of MPM from imaging studies include nodular pleural thickening, pleural plaques, and pleural effusion. We aimed to present a case whose thoracoscopy showed no pathological evidence on pleural surfaces while a computed tomography of the chest showed pleural thickening, and PET showed no pathological FDG uptake but resulted in bilateral chylothorax whose mediastinal lymph node sampling was reported as MPM.

## 2. Case Presentation

**Figure 1:**
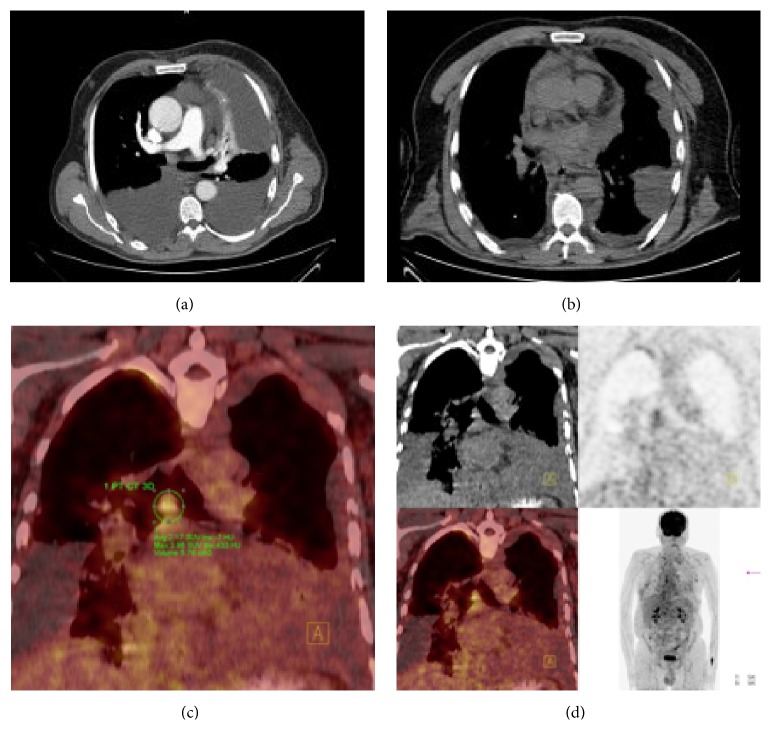
(a) A bilateral pleural effusion is noted in the CT obtained during first presentation. (b) A CT scan shows no nodular formation in the pleural surfaces following the drainage of pleural effusion. (c) A coronal PETCT image at the level of the mediastinal nodes. (d) PET-CT images are shown.

**Figure 2:**
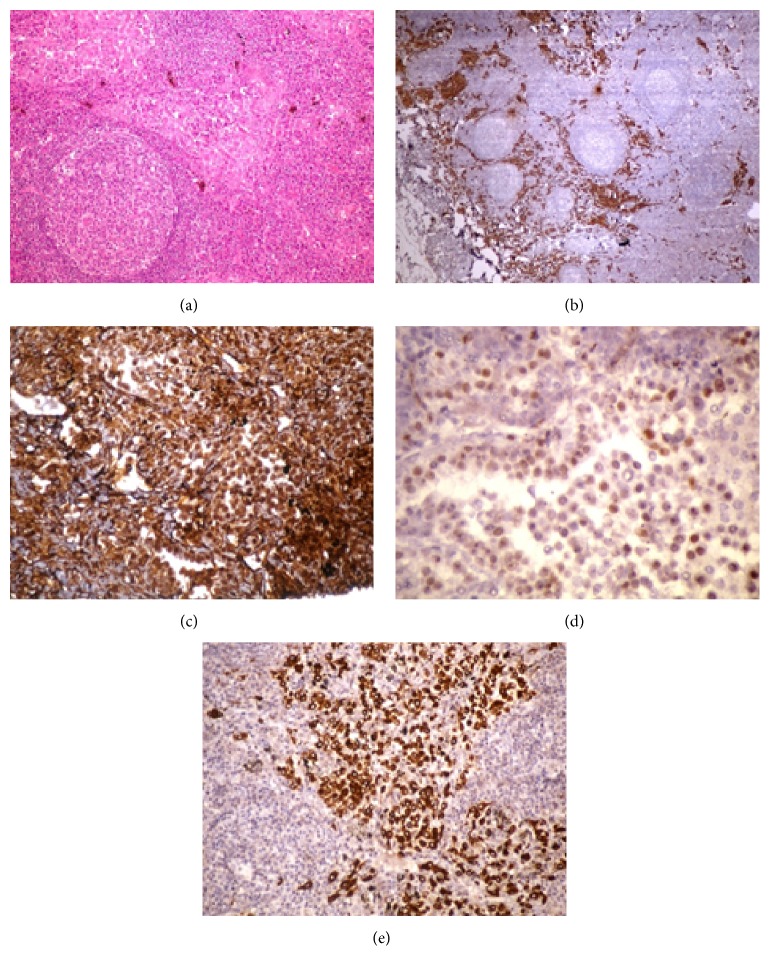
(a) Tumoral infiltration was seen between the lymphoid follicles. Tumor cells are medium large cells with large eosinophilic cytoplasm, oval round vesicular nucleus, and distinct nucleolus (HE × 200). (b) Cytoplasmic staining in tumor cells with immunohistochemical Pan-CK stain. Pan-CK × 100. (c) Cytoplasmic and nuclear staining in tumor cells with immunohistochemical Calretinin stain. Calretinin × 400. (d) Nuclear staining in tumor cells by immunohistochemical WT-1 stain. WT-1 × 400. (e) Cytoplasmic staining of tumor cells with immunohistochemical CK 5/6 stain. CK 5/6 × 200.

A 60-year-old male presented to our clinic with a complaint of shortness of breath. A computed tomography (CT) showed bilateral pleural effusion (Figure 1(a)) while CT analysis showed no thickening of pleural surfaces and nodular formation. Thoracentesis showed a chylous effusion. A biochemical analysis of the pleural fluid sample showed a triglyceride level of 1228 mg/dL and a cholesterol level of 149 mg/dL. The patient received bilateral thoracic drainage after withdrawal of oral nutrition, and he was initiated on a protein-rich and fat-poor parenteral nutrition. A new CT, which was repeated to prevent overlooking any potential lesions in the parenchyma after drainage of effusion, showed no thickening of pleural surfaces or any nodular formation (Figure 1(b)). Similarly, PET-CT showed no pathological FDG uptake (Figures 1(c)-1(d)). During monitoring, a surgery was planned at day 10 since drainage was reduced to less than 500 cc but persisted above 250 cc. The patient received 200 cc olive oil by nasogastric tube 2 hours before the surgery. He underwent right thoracotomy, and ductus thoracicus was identified between azygos vein and esophagus during the operation. It was followed by mass ligation just above the diaphragm. No pathology was observed in parietal and visceral pleura during the surgery suggesting mesothelioma. Specimens were collected from the mediastinal lymph nodes and sent to the pathology laboratory. A microscopic examination of the lymph node showed tumoral infiltration in a scattered pattern with layers under the capsule and between lymphoid follicles. Tumor cells are medium large cells with large eosinophilic cytoplasm, oval round vesicular nucleus, and distinct nucleolus (Figure 2(a)). The immunohistochemical analysis showed diffuse and strong staining with Pan-CK (Figure 2(b)), Calretinin (Figure 2(c)), WT-1 (Figure 2(d)), vimentin, CK7, HBME-1, and D2-40 and focal staining with CK5/6 (Figure 2(e)), CEA, and EMA in tumor cells. No immune reaction was observed in tumor cells with CD68, TTF-1, Napsin-A, Melan-A, S-100, HMB45, LCA, Heppar-1, PLAP, and CK20 used for differential diagnosis. Based on these findings, the patient was diagnosed with metastasis of malignant mesothelioma. A recent CT which was performed at postoperative month 1 due to persistence of patient's complaints showed diffuse nodular formation. A pathological analysis of the lymph node reported MPM. Following a consultation with the medical oncology, a chemotherapy regimen was prescribed; however, the patient was unable to receive the treatment due to his impaired general condition.

## 3. Discussion

Clinically patients with chylothorax develop progressive dyspnea and tachypnea. Auscultation reveals decreased respiratory sound on the relevant side. Our patient presented to our clinic with such complaints. Lymphoma is the most common cause of nontraumatic chylothorax [1]. Computed tomography is not efficient in locating the site of chyle leakage but is helpful in identifying the location of a mediastinal or thoracic lesion [3]. Fifty percent of patients with Hodgkin's lymphoma have lymphadenopathy in their thorax [4]. Puncture of pleural effusion common in most of the patients is the first diagnostic method for diagnosis of MPM. A cytologic examination of the pleural fluid allows establishment of diagnosis in 20% to 50% of patients. A percutaneous pleural biopsy may help in the diagnosis of one-third of the patients. Treatment of chylothorax initially requires drainage of the chylous in the pleural space. However, surgical treatment is recommended in case of inefficient drainage and development of nutritional complications despite conservative management [1, 5]. The mortality of chylothorax was around 50% in the absence of surgery. The landmark in the treatment of chylothorax has been the initial successful treatment of a patient by ligation of ductus thoracicus in 1948 by Lampson [6].

Malignant pleural mesothelioma (MPM) is a rare condition, most commonly caused by exposure to asbestos, with increasing incidence worldwide. It generally occurs in the 5th to 7th decades of life, and 70–80% of patients are males. It is more common in men, which is mainly attributed to occupational exposure. The most common presenting complaints are shortness of breath and chest pain. One third of patients have shortness of breath without chest pain [7]. Our patient also had only a complaint of shortness of breath. Imaging has an important role in evaluating the response (especially in terms of resectability), treatment planning, monitoring, and diagnosis of patients with MPM. The imaging modalities used for diagnosis and treatment of MPM include X-ray, computed tomography (CT), magnetic resonance imaging (MRI), and positron emission tomography (PET). In BT, any evidence of unilateral pleural effusion, circumferential diffuse or pleural thickening, and shrinkage in hemithorax suggests MPM [8–10]. Our patient had no involvement of pleural surfaces during first presentation. There are some histological challenges in the diagnosis. A major challenge is the differentiation of malignant mesothelioma from reactive mesothelial cells. The same challenge exists for differentiation from lung adenocarcinomas [11].

Although several methods are available for treatment of MPM, there exists a consensus only for chemotherapy. The prognosis is usually poor in malignant mesotheliomas. A mean survival time of 4–12 months has been reported. The prognosis is better in female patients whose symptoms manifest in a period more than six months in younger ages without chest pain and involvement of visceral pleura [12].

In conclusion, MPM should be considered in patients presenting with particularly unilateral pleural effusion, pleural thickening, and chest pain. We believe that in our patient, the combination of MPM and bilateral chylothorax was associated with the rupture of the ductus thoracicus as a result of the invasion of the tumor in the mediastinal pleura into the mediastinum and ductus thoracicus.
